# Weaker SARS-CoV-2 vaccine responses in nonalcoholic fatty liver disease with advanced liver fibrosis

**DOI:** 10.1016/j.jvacx.2023.100359

**Published:** 2023-10-12

**Authors:** David Hakimian, Johnny Amer, Alaa Jammal, Asher Shafrir, Yael Milgrom, Mohammad Masarowah, Wadi Hazou, Yuval Ishay, Ashraf Imam, Adi Francis, Abed Khalaileh, Rifaat Safadi

**Affiliations:** a Hadassah Medical Center, Liver insitute, Hadassah-Hebrew University Medical Center, Israel; b Hadassah Medical Center, Department of Surgery, Jerusalem, Israel; c Hadassah Medical Center, Cardiac Care Unit, Holy Family Hospital, Bar-Ilan University, Nazareth, Israel

**Keywords:** Pfizer's mRNA SARS-CoV-2 vaccine, Serum SARS-CoV-2 spike immunoglobulins, NAS score, Liver fibrosis stage, Fibroscan

## Abstract

**Background:**

SARS-CoV-2 vaccine responses that could harbor potential risks to chronic liver diseased patients.

**Aims:**

To assess immune response following Pfizer's SARS-CoV-2 vaccine in patients with different liver fibrosis severities of nonalcoholic fatty liver disease (NAFLD).

**Methods:**

Clinical and histological (NAS-score and fibrosis stage) characteristics of NAFLD patients before vaccine were correlated with serologic vaccine responses of two doses of the BNT162b2. Serum SARS-CoV-2 spike immunoglobulins (anti-S) were assessed on day seven following immunization (Liaison assay).

**Results:**

The mean-age of patients (n = 157) was 56.9 ± 13.2 years (46.5 % males). 94.8 % had a positive response (anti-S levels ≥ 19 AU/ml). The anti-S cutoff of 200 AU/ml used to separate strong *vs.* weak responses. A strong response (anti-S titers ≥ 200 AU/ml) was observed in 93/157 (59.2 %) patients with a mean-age of 53.1 ± 13.8 years (45.2 % males). A weak response (anti-S titers < 200 AU/ml) was observed in 64/157 (40.8 %) cases with a mean-age of 62.3 ± 10.2 years (p < 0.0001). The strong response subgroup had lower metabolic comorbidities, including glucose hemostasis, hypertension, and dyslipidemia (p < 0.04). Moreover, the strong response subgroup had fibrosis stages F0-F2 (75.3 % *vs.* 56.3 %) and lower rates of advanced stages F3-F4 (24.7 % *vs.* 43.8 %). The F0-F2 subgroups had significantly higher rates of strong responses than the F3-F4 stages. The anti-S ≥ 200 and anti-S ≥ 400 AU/ml response achieved in 66 % and 36.8 % of the F0-F2 population was significantly higher than the 45.1 % (p = 0.006) and 23.5 % (p = 0.05) in the F3-F4 population, respectively. The Fib-4 calculations and Fibroscan evaluations were consistent with histologic fibrosis assessment.

**Conclusion:**

Advanced liver fibrosis (assessed by histology, Fib-4, or Fibroscan) is a risk factor for lower response to Pfizer's BNT162b2 vaccine, and patients should be prioritized for the vaccine booster against SARS-CoV-2.

## Highlights

• Advanced liver fibrosis scores impaired Pfizer's BNT162b2 vaccine response.
• A strong vaccine response was observed in cases with a mean-age of 53.1 ± 13.8.
• A weak vaccine response was observed in cases with a mean-age of 62.3 ± 10.2.
• The F0-F2 subgroups had a strong response than the F3-F4 stages.

## Introduction

Coronavirus disease (SARS-CoV-2) was reported by the end of 2019. Since the first wave of the SARS-CoV-2 pandemic, striking mainland China [1], health organizations emphasized the urgent call and need for an efficient vaccination program [1]. The race for vaccinations began promptly with the development of various vaccines [2]. Currently, several vaccines are approved, with a few more on the companies' [3] pipeline, in the late stages of clinical trials.

On December 11, 2020, the US Food and Drug Administration (FDA) issued an Emergency Use Authorization (EUA) for the Pfizer-BioNTech SARS-CoV-2 (BNT162b2) vaccine (Pfizer, Inc; Philadelphia, Pennsylvania), a lipid nanoparticle-formulated, nucleoside-modified mRNA vaccine encoding the prefusion stabilized, membrane-anchored SARS-CoV-2 full-length spike glycoprotein of SARS-CoV-2. Vaccination with the Pfizer-BioNTech SARS-CoV-2 vaccine consists of two doses (30 μg, 0.3 mL each) administered intramuscularly, 21 days apart. The vaccine demonstrates efficacy in clinical and real-world data. A two-dose regimen of BNT162b2 conferred 95 % protection against SARS-CoV-2 occurrence in persons 16 years of age or older. Israel initiated a vaccination program on December 19, 2020. >93 % of the eligible population (SARS-CoV-2 naïve subjects above the age of 16 and recently above 12 years) received at least one dose, and 89 % completed both doses of the Pfizer vaccine [4]. The Israeli vast vaccination experience suggests that the BNT162b2 mRNA vaccine is effective for a wide range of SARS-CoV-2-related outcomes, a finding consistent with that of the randomized trial [5].

Although vaccine efficacy is defined as protection from SARS-CoV-2 occurrence and not serologic response, it is commonly accepted that failure cases do not achieve a serologic response. Serum antibody levels to determine vaccine efficacy were measured in non-SARS-CoV-2 vaccinees [6]. Thus, serology assessment is well validated in SARS-CoV-2 vaccinees [7] and might serve as a simple biomarker for a new future strategy. Accordingly, post-vaccine serologic surveillance for defining the high-risk population is mandatory for an upcoming approach to booster vaccines. The SARS-CoV-2 serology became a routine test worldwide and was adopted in some circumstances for clinical judgment by governmental health guidelines in Israel.

The historical non-SARS-CoV-2 vaccines have shown that several factors are associated with vaccine effectiveness [8], including intrinsic host factors (age, gender, and comorbidities) and extrinsic factors (preexisting immunity, microbiota, infections, genetics, and antibiotics). The argument that severe liver disease patients have a lower response rate to vaccines is well-established [9]. In fact, patients with cirrhosis or chronic hepatitis C infection are shown to have less seroconversion following a single dose of the hepatitis A virus vaccine compared to controls [10]. Patients with chronic hepatitis B displayed lower mean titers than healthy controls six months following the hepatitis A virus vaccine. The risk factors for SARS-CoV-2 mRNA vaccines and particularly Pfizer-BioNTech's decreased immunogenicity in liver disease patients are unknown. Vaccinations are more efficient in the early stage of liver disease, avoiding the immunity defect in cirrhosis [11]. As age was identified as a leading factor affecting vaccines response, a booster dose was suggested for older people. On July 30, 2021, the administration of a third (booster) dose of the BNT162b2 messenger RNA vaccine (Pfizer-BioNTech) was approved in Israel for persons who were 60 years or older and who had received a second dose of the vaccine at least five months earlier [12]. Due to the lack of relevant literature regarding the efficacy of mRNA vaccines in liver diseases and particular NAFLD, we aimed to estimate the serologic response and protection of the Pfizer-BioNTech vaccine in patients with NAFLD. Identification of liver risk factors might affect priority considerations for vaccine boosting.

## Patients and methods

### Local strategy

Patients in the Hadassah Medical Organization (HMO) liver unit were actively encouraged to receive both vaccines as part of the national vaccination policy to overcome vaccine hesitation. To increase vaccination compliance, serologic assessment of SARS-CoV-2 spike immunoglobulins (anti-S) was offered at least one week after the second vaccination. Non-responders were informed to keep strict protection against SARS-CoV-2.

## Study design and patients

This is a retrospective, single-center study designed at HMO, the largest hospital in the Jerusalem area. Adult male and female patients (≥18 years old) with pre-and *peri*-vaccination (up to 3 months after the second vaccine dose) with a diagnosis of NAFLD by liver biopsy were included. The current pilot included the cases that underwent anti-S evaluation at least one week after the second vaccination. Patients excluded were those with additional liver etiologies, alcohol consumption that exceeded the average acceptable daily amounts (up to 25 g a day in women and 35 g in men), patients treated with immune suppressors or having other significant comorbidities, such as malignancies or an acute disease at the time of vaccination. Patients with a history of SARS-CoV-2 infection or positive anti-N serology were excluded from the study. Also excluded were cases with missing information such as laboratory tests, vaccination dates and lack of biopsy.

Anti-S IgG was measured using the DiaSorin's LIAISON® kit [13] at least one week after the second vaccination. Serum anti-S levels of ≥ 19 AU/ml were considered good vaccine response, serum anti-S levels of < 12 AU/ml were considered vaccine failure, and an equivocal response refers to serum anti-S levels ≥ 12 but < 19 AU/ml. The anti-S DiaSorin's LIAISON® kit gave a scale from undetectability up to 400 AU/ml. Higher anti-S levels were recorded as > 400 AU/ml.

Additional clinical and laboratory data retrieved by the HMO Information Technology System included the participant's age, gender, habits such as smoking, illicit drugs usage, and alcohol consumption. Other parameters were anthropometric measures such as Body mass index (BMI), and pre-vaccination blood tests. Biochemistry that includes aspartate aminotransferase (AST), alanine transaminase (ALT), gamma-glutamyl transferase (GGT), alkaline phosphatase (ALP), albumin, total bilirubin and other biochemical measurements. Total blood count values included platelets (PLT), white blood cells (WBC), hemoglobin (Hgb), and clotting factors (international normalized ratio (INR), partial thromboplastin time (PTT) and Fibrinogen). The patient's pathology report was formatted to the NAS scoring system. The NAS is defined as the sum of three equally weighted parameters: steatosis (0–3), lobular inflammation (0–3), and hepatocellular ballooning (0–2), achieving a total score of (0–8) [14]. The pathology report was formatted to fibrosis levels (0–4) based on the NASH Clinical Research Network (CRN). Calculated pre-vaccination Fib-4, elastography, and CAP readings were also collected when available.

The study protocol (including the Liver Unit local strategy) conformed to the ethical guidelines of the 1975 Declaration of Helsinki and was approved by the local HMO Ethics Committee (Trial registration number: 1000-20-HMO). The local ethics committee waived informed consent since the vaccine administered and testing the serologic response were standard of care practice. All authors had access to the study coded data (without personal details), reviewed, and approved the final manuscript.

### Statistical analysis

For descriptive analysis, we used counts and percentages for categorical variables. Continuous variables are summarized as means and standard deviation (SD). T-test was used to compare means of continuous variables. A P-value of<0.05 was considered statistically significant in all analyses. Statistical analysis was performed using R software (R Development Core Team, 2018, PBC, Boston, MA).

## Results

### Characterization of the study population

**Fig. 1:**
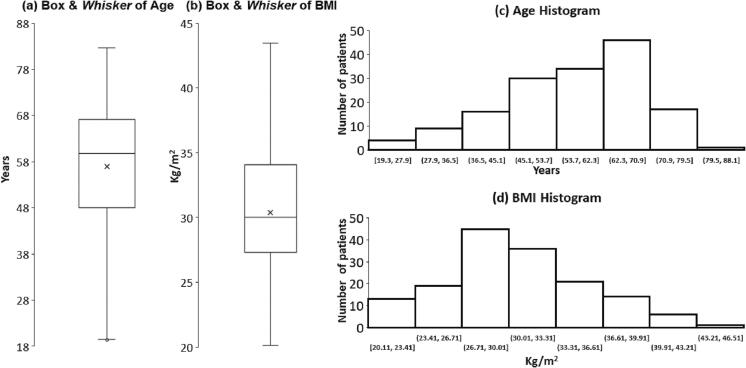
Age and BMI Box and Whisker and histogram. Box and Whisker and histogram showing age (Fig. 1a and 1c, respectively) and BMI (Fig. 1b and 1d, respectively). The main age subgroups were 53.7–70.9 years and 26.7–36.6 kg/m^2^ for BMI.

The study group included 157 patients who fulfilled the inclusion and exclusion criteria. Mean age was 56.9 ± 13.2 years, 46.5 % were males, average BMI was 30.4 ± 5.1, 40.1 % were Jews and 59.9 % Arabs, social alcohol consumption was reported in 2.3 %, and 16.8 % were active or past smokers (Table 1). Box and Whisker and histogram of age and BMI, shown in Fig. 1, demonstrate that the main subgroups were 53.7–70.9 years for age and 26.7–36.6 kg/m^2^ for BMI.

**Table 1:** Characterization of the 157 patients in the study group who fulfill the criteria (Total), and two subgroups according to anti-S titers, strong responders (anti-S ≥ 200 AU/mL, N = 93) and weak responders (anti-S < 200 AU/mL, N = 64). Table 1a shows the patient's demographics, and Table 1b shows background metabolic manifestations. A P-value of ≤ 0.05 is considered significant.

Table-1a				
	**Total** (N = 157)	**anti-S ≥ 200** (N = 93)	**anti-S < 200** (N = 64)	**P**
				
**Age** (years)	56.9 ± 13.2	53.1 ± 13.8	62.3 ± 10.2	**<0.0001**
**BMI**	30.4 ± 5.1	30.4 ± 5.1	30.5 ± 5.1	0.446
**Male** (%)	46.5 %	45.2 %	48.4 %	0.344
**Jew** (*vs.* Arab)	40.1 %	35.5 %	46.9 %	0.077
**Alcohol** (%)	2.3 %	2.2 %	2.4 %	0.474
**Smoking** (%)	16.8 %	19.7 %	12.8 %	0.168

Table-1b				
	**Total** (N = 157)	**anti-S≥200** (N = 93)	**anti-S<200** (N = 64)	**P**
**DM/IFG**	69.0 %	61.6 %	78.6 %	**0.020**
**Fasting Glucose** (mmol/L)	8.3±13.2	6.5±3.0	10.7±19.6	**0.038**
**HbA1c** (%)	6.0±1.5	6.0±1.8	6.0±1.0	0.485
**GLA's**	45.8 %	39.5 %	52.5 %	0.121
**Hypertension**	49.6 %	40.3 %	62.5 %	**0.009**
**Anti-hypertensive**	46.3 %	31.0 %	62.5 %	**0.002**
**Dyslipidemia**	88.0 %	85.4 %	90.9 %	0.211
**TG** (mmol/L)	1.6±1.2	1.6±0.7	1.6±1.6	0.487
**LDL**(mmol/L)	2.5±0.9	2.6±0.9	2.3±1.0	0.108
**HDL**(mmol/L)	1.7±5.1	2.2±7.1	1.2±0.3	0.173
**Statins & Fibrates**	51.8 %	39.5 %	65.0 %	**0.010**

**Fig. 2:**
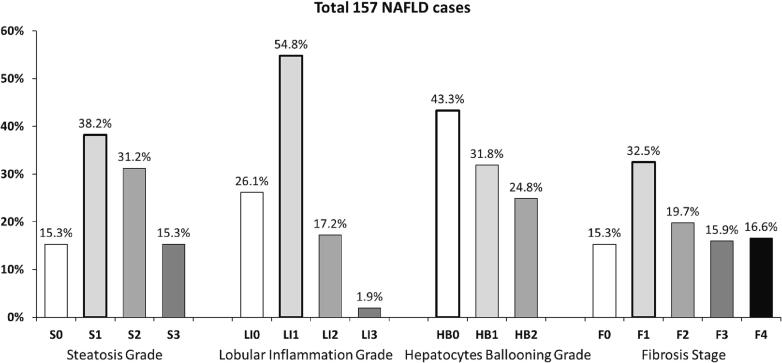
Histologic NAS grading and fibrosis scoring. Histologic NAS grading and fibrosis scoring of 157 cases were assessed blindly. Each column shows the percentage of patients for each parameter. Steatosis was graded S0, S1, S2, and S3. Lobular inflammation was graded LI0, LI1, LI2 and LI3. Hepatocyte ballooning was graded HB0, HB1, and HB2. CRN fibrosis was scored F0, F1, F2, F3, and F4.

All 157 cases had been referred to liver biopsy to evaluate the staging and grading of fatty liver disease documented by pre-biopsy noninvasive tools (abdominal ultrasound, CT, MRI, and Fibroscan) and to assess other liver competing etiologies (excluded in this study). Five cases underwent liver biopsy up to 3 months after the second vaccine, and the other 152 had a pre-vaccination biopsy. The mean biopsy to vaccine interval was 3.2 ± 3.6 years (from 16.3 pre-vaccination to 0.4 years after the second vaccine). Histologic NAS grading and fibrosis scoring were assessed blindly and are summarized in Fig. 2. Steatosis was absent (S0) in 15.3 % of cases despite clinical and hepatic imaging diagnoses. However, grade 1 steatosis (S1) was observed in 38.2 %, S2 in 31.2 %, and S3 in 15.3 % cases. Lobular inflammation was absent (LI0) in 26.1 %, LI1 was present in 54.8 %, LI2 in 17.2 %, and LI3 in 1.9 % of cases. Most of the patients (43.3 %) had no hepatocyte ballooning (HB0). However, HB1 was found in 31.8 % and HB2 in 24.8 % of cases. CRN fibrosis score was F0 in 15.3 %, F1 in 32.5 %, F2 in 19.7 %, F3 in 15.9 % and F4 in 16.6 % of cases (32.5 % of cases are with advanced fibrosis F3-F4).

**Fig. 3:**
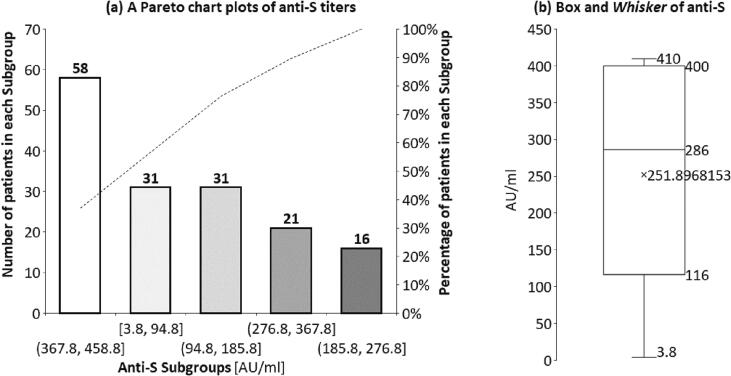
Pareto chart plots and Box and Whisker of Anti-S IgG. Anti-S IgG was measured at least one week following the second vaccine shot, using the DiaSorin's LIAISON® kit. Of the 157 cases, 152 (94.8 %) achieved a positive response (anti-S levels ≥ 19 AU/ml). The distribution of serum anti-S concentrations demonstrated by Pareto chart plots (Fig. 3a) and Box and Whisker (Fig. 3b) revealed 62 cases (39.5 %) with anti-S titers < 200 AU/ml. A P-value of ≤ 0.05 is considered significant.

Anti-S IgG was measured at least one week following the second vaccine shot, using the DiaSorin's LIAISON® kit. The vaccine to serology test intervals were 43.6 ± 35.3 days, ranging from 7 to 173 days post-second vaccine. Except for five case (3.2 %) with serologic vaccine failure (anti-S levels < 12 AU/ml), all 152 (94.8 %) patients achieved a positive response (anti-S levels ≥ 19 AU/ml). The distribution of serum anti-S concentrations by Pareto chart plots (Fig. 3a and 3b) revealed 62 cases (39.5 %) with anti-S titers < 200 AU/ml. The other 95 cases (60.5 %) achieved anti-S titers ≥ 200 AU/ml. Based on earlier experience [15] and current Pareto chart plots, 200 AU/ml anti-S cutoff differentiates between strong and weak responses.

## Correlation of vaccine response by anti-S levels and liver pathology

**Fig. 4:**
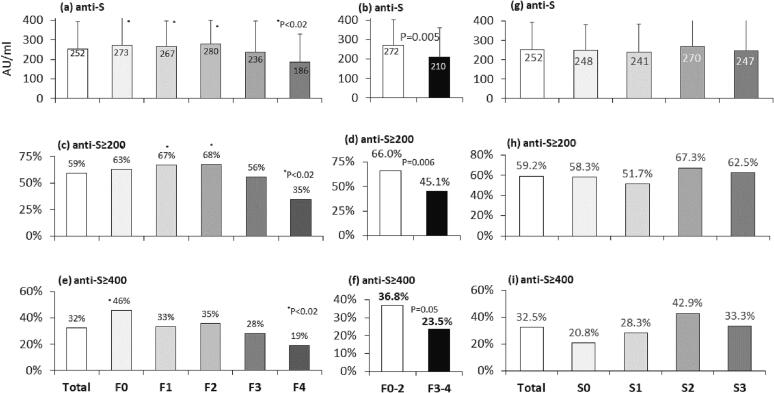

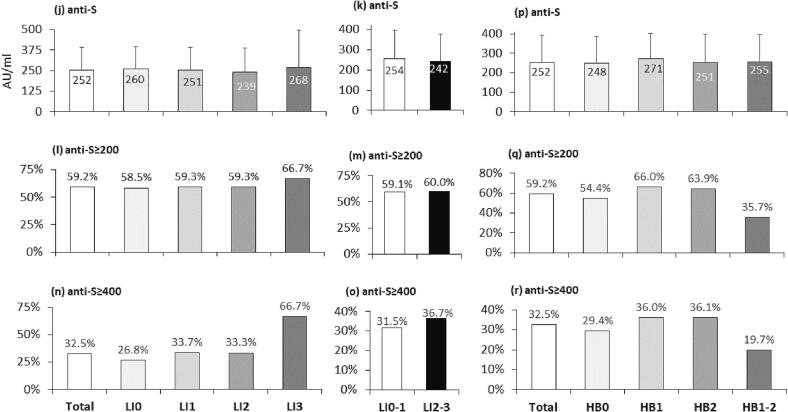
Levels of anti-S titers according to histologic fibrosis stages and NAS grades. Levels of anti-S titers were evaluated according to histologic fibrosis stages and grades of the three NAS components. steatosis (S), lobular inflammation (LI), hepatocyte ballooninr (HB). For each parameter, the figure presents the mean anti-S titer per grade (AU/ml, Fig. 4a, 4b, 4g, 4j, 4k, 4p) and the percentage of patients exceeding the anti-S thresholds of 200 AU/ml (Fig. 4c, 4d, 4h, 4l, 4m, 4q) or 400 AU/ml (Fig. 4e, 4f, 4i, 4n, 4o, 4r). A P-value of ≤ 0.05 is considered significant.

We next correlated levels of anti-S titers to histologic characteristics of liver fibrosis stages and the three components of NAS scoring (Fig. 4a − 4r). Mean anti-S titers and percent of cases exceeding anti-S thresholds (anti-S ≥ 200 and anti-S ≥ 400 AU/ml) were assessed for each histologic parameter according to its severity. The 200 AU/ml anti-S threshold was used based on earlier experience [15] and current Pareto chart plots. The anti-S threshold of 400 AU/ml was the upper limit of our kit. The mean absolute anti-S titers were similar in low fibrosis stages F0, F1, and F2; however, they were significantly higher than titers in F4 (Fig. 4a). Titers from F3-scored patients showed a trend for reduction, but the change was not significant. Combined low fibrosis groups F0-F2 (Fig. 4b) compared to combined advanced fibrotic groups F3-F4 showed a significant reduction of anti-S titers in advanced fibrosis from 271.9 ± 130.9 to 210.3 ± 151.7 AU/mL (p = 0.005). A similar pattern was observed when the results were analyzed according to both anti-S thresholds (Fig. 4c − 4f). Anti-S thresholds of ≥ 200 and ≥ 400 AU/ml were achieved in 66 % and 36.8 % of the low fibrosis groups, compared to 45.1 % (p = 0.006) and 23.5 % (p = 0.05) in advanced fibrosis groups, respectively (Fig. 4d, 4f). Anti-S titers showed comparable levels to steatosis (S, Fig. 4g − 4i), lobular inflammation (LI, Fig. 4j − 4o), and hepatocyte ballooning (HB, Fig. 4p − 4r). There was a statistically significant association between moderate steatosis (S2) and a good vaccine response (S, Fig. 4h, 4i).

## Correlation of liver pathology and vaccine response by anti-S levels

To confirm the association of advanced fibrosis with lower anti-S titers, we correlated histologic findings of NAFLD with an anti-S titer threshold of 200 AU/ml. The strong responder (anti-S titers ≥ 200 AU/ml) and weak responder (anti-S titers < 200 AU/ml) subgroups included 93/157 (59.2 %) and 64/157 (40.8 %) cases, respectively, with a mean age of 53.1 ± 13.8 *vs.* 62.3 ± 10.2 years (p < 0.0001), 45.2 % *vs.* 48.4 % males (p = 0.077), respectively. BMI was 30.4 ± 5.1 in both groups; Jews and Arabs were 45.2 % and 54.8 % *vs.* 46.9 % and 53.1 %, respectively. Social alcohol consumption was reported in 2.2 % *vs.* 2.4 % cases, and 19.7 % *vs.* 12.8 %, respectively, were active or past smokers (Table 1a).

**Fig. 5:**
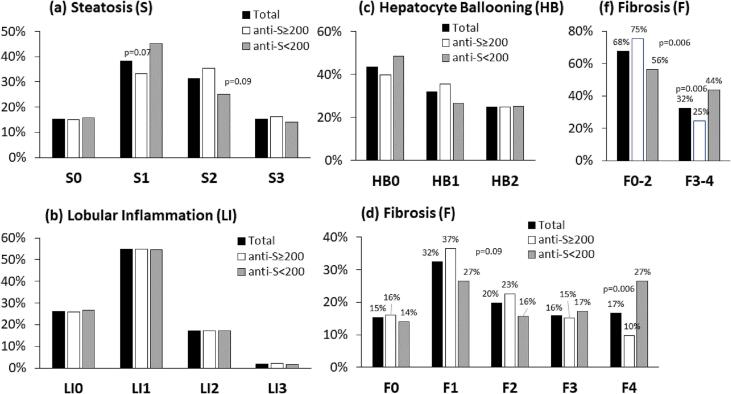
Prevelance of strong and the weak responders in each liver fibrosis score and NAS grade. Percentage of cases per fibrosis score and NAS grade (for the three NAS components) shown in the whole group (black bars), in the strong responders (anti-S titers ≥ 200 AU/ml, white bars), and the weak responders (anti-S titers < 200 AU/ml, gray bars). For fibrosis stages, results are shown for each stage (Fig. 5d) and low (F0-F2) *vs.* advanced (F3-F4) fibrosis (Fig. 5f). A P-value of ≤ 0.05 is considered significant.

Strong *vs.* weak responder subgroups were divided according to the stage or grade of each histologic parameter (Fig. 5). The three NAS components showed comparable results in strong *vs.* weak responders. Both subgroups revealed a similar degree of steatosis distribution (Fig. 5a), lobular inflammation (Fig. 5b), and hepatocyte ballooning (Fig. 5c). However, a significant change in the pattern repeated when strong *vs.* weak responses were correlated to fibrosis stages (Fig. 5d, 5f). The low fibrosis stage F1 patients included 36.6 % of those who achieved titers ≥ 200 AU/ml *vs.* 26.6 % with titers < 200 AU/ml (tend to be higher, p = 0.09). However, the cirrhosis stage F4 patients included only 9.7 % of those achieving titers ≥ 200 AU/ml *vs.* 26.6 % with titers < 200 AU/ml (significantly lower, p = 0.006). Combining low fibrosis groups F0-F2 and comparing them to advanced fibrosis groups F3-F4 showed more substantial differences. Fig. 5f shows that 75.3 % of the strong responders and 56.3 % of the weak responders (p = 0.006) had low fibrosis stages F0-F2 compared to 24.7 % and 43.8 % (p = 0.006) in the advanced fibrosis stages F3-F4, respectively.

## Correlation of metabolic outcomes and laboratory liver injury according to vaccine response by anti-S levels

In addition to fibrosis predicting the quality of vaccine response, the strong responders had better metabolic outcomes than the weak responders (Table 1b). The strong response subgroup showed lower rates of diabetes mellitus and impaired fasting glucose (61.6 % *vs.* 78.6 % of cases, p = 0.02) and lower mean serum fasting glucose (6.5 ± 3.0 *vs.* 10.7 ± 19.6 mmol/L, p = 0.038) than weak responders. However, both subgroups had the same percentage of patients treated with glucose lowering agents (GLA’s), achieving a similar control according to HBA1c levels. The strong response subgroup also had a lower prevalence of hypertension (40.3 % *vs.* 62.5 %, p = 0.009) and lower use of hypertensive lowering agents (31 % *vs.* 62.5 % of cases, p = 0.002). Although dyslipidemia prevalence and blood levels were similar, the strong response subgroup required a lower use of statins and fibrates (39.5 % *vs.* 65 % of cases, p = 0.01), respectively.

A similar pattern was observed in manifestations of advanced liver disease (Table 2a and b). The strong responders had higher white blood counts (6.8 ± 2.2 *vs.* 6.2 ± 2.1 K/uL, p = 0.053), hemoglobin levels (13.7 ± 1.7 *vs.* 12.9 ± 1.8 g/dl, p = 0.004), platelet counts (226.1 ± 87.2 *vs.* 196.8 ± 112.3 *10^9^/L, p = 0.04) and serum albumin levels (41.9 ± 5.4 *vs.* 38.7 ± 11.1 gr/L, p = 0.02) than the weak responders. However, the strong responders had lower levels of INR (1.0 ± 0.1 *vs.* 1.1 ± 0.2, p = 0.004) and GGT (72.6 ± 71.5 *vs.* 149.3 ± 284.9 U/L, p = 0.022) than the weak responders. Creatinine showed a trend of improvement in the strong responders (67.6 ± 16.4 *vs.* 74.7 ± 32.7 μmol/L, p = 0.056). Fibrinogen, PTT, alanine and aspartate aminotransferases, alkaline phosphatase, sodium, total bilirubin, vitamin D, glycosylated hemoglobin, triglycerides, and cholesterols did not show a significant difference between the two groups.

## Correlation of noninvasive liver injury and vaccine response by anti-S levels

To validate the finding that advanced liver fibrosis is associated with lower anti-S titers using noninvasive tools, we analyzed available pre-vaccination Fib-4 values from 131 patients and Fibroscan results from 72 patients according to the anti-S titers threshold of 200 AU/ml (Table 3).

Mean Fib-4 values were 2.4 ± 2.6 in all 131 patients, reflecting the indication for liver biopsy. Mean Fib-4 values were significantly lower (1.7 ± 1.5 *vs.* 3.4 ± 3.3, p < 0.0001) in the strong response group (anti-S ≥ 200 AU/mL, N = 77) compared to the weak response group (anti-S < 200 AU/mL, N = 54). These data were in accord with lower rates of advanced fibrosis (defined as Fib-4 < 2.67) in 11.7 % *vs.* 40.7 %, p < 0.0001, respectively (Table 3a).

The mean pre-vaccination Fibroscan elastography value of 11.3 ± 9.3 kPa, obtained from 72 cases, is relatively high, justifying the performed biopsy. However, mean elastography values were significantly lower (9.2 ± 4.2 *vs.* 13.6 ± 12.4, p = 0.016) in the strong response group (anti-S ≥ 200 AU/mL, N = 41) compared to the weak response group (anti-S < 200 AU/mL, N = 31). Also, there were lower rates of advanced fibrosis, defined as E > 8.5 kPa (in 47.6 % *vs.* 65.8 %, p = 0.052) or E > 12 kPa (in 23.8 % *vs.* 42.1 %, p = 0.041), in the strong *vs.* weak response groups, respectively (Table 3b). Although mean CAP readings did not show differences between the response groups, the advanced steatosis (S3) defined as > 331 dB/m was significantly lower in the strong responders as compared to the weak responders (22 % *vs.* 41.9 %, p = 0.035), respectively (Table 3c).

## Discussion

Vaccines are expected to play a crucial role in ending the SARS-CoV-2 pandemic. Patients on B- cell depleting therapy such as ocrelizumab [16] and rituximab [17] and solid organ recipients [18], [19] have an attenuated vaccine response. However, there is only limited information regarding the efficacy of the currently available SARS-CoV-2 mRNA vaccines in patients with NAFLD. NAFLD is a chronic and increasingly common liver disorder among adults in western countries. NAFLD encompasses a heterogeneous spectrum of diseases, characterized as ≥ 5 % liver steatosis with no evidence of injury to hepatocytes and no evidence of fibrosis. Nonalcoholic steatohepatitis (NASH) [20], [21] is defined as ≥ 5 % liver steatosis accompanied by inflammation and injury to hepatocytes with or without fibrosis, although fibrosis is typically observed. NASH is associated with an increased risk of cirrhosis, hepatocellular carcinoma, and liver‐related mortality, especially when fibrosis is present [22]. The advancing liver fibrosis stage exponentially increases the risk of liver‐related mortality [23].

This is the first study that evaluates the efficacy of Pfizer's BNT162b2 vaccine in NAFLD patients associating the anti-S IgG serology data and liver severities based on histological staging and grading. The high efficacy of the Pfizer-BioNTech vaccine has been demonstrated in a controlled study and big real-world data [24]. However, studies that attempt to find risk factors for vaccine ineffectiveness are scarce. While the gold standard for vaccine ineffectiveness is SARS-CoV-2 infection, antibody titers can be a good marker to predict vaccine response. Serologic assays have been developed and validated to accurately detect anti–SARS-CoV-2 nucleocapsid antibodies, which are elicited by naturally acquired infection but not by a SARS-CoV-2 spike protein-based vaccination [25]. Furthermore, subjects with higher serum S IgG titers post SARS-CoV-2 disease [26] or among vaccinated individuals [27] were less likely to have a subsequent infection. Thus, a serology-based assay is a reliable surrogate for the evaluation of vaccine efficacy.

The current study showed that advanced liver fibrosis scores impaired Pfizer's BNT162b2 vaccine response. However, none of the NAS elements affected the response (including steatosis, lobular inflammation, and ballooning). Low serologic response in advanced histologic fibrosis was associated with significantly higher age, metabolic comorbidities, and increased blood markers of liver injury and advanced fibrosis. The mean age of the weak responders in the current study was significantly higher than that of the strong responders (P < 0.0001). However, gender, origin, alcohol, and smoking were similar (Fig. 1, Table 1). This fact is comparable to other non-SARS-CoV-2 vaccines, where diminished antibody response in older people and reduced vaccination longevity [28] have been reported. A third of our population (Fig. 2) had advanced fibrosis (F3-F4), compatible with known distributions in NAFLD biopsy populations. Here we report an inverse association between liver fibrosis severities in NAFLD patients and Pfizer's BNT162b2 vaccine immunogenicity, demonstrating that advanced fibrosis, but not NAS grading, correlated with lower post-vaccination anti-S IgG titers (Fig. 4&5). Advanced fibrosis and cirrhosis have been associated with immune impairment of innate and acquired immunity, leading to immunodeficiency [29].

Multiple factors may contribute to these changes in immune activity. T cells were shown to participate in immune senescence [30]. However, the role of B cells remains unclear. Recent findings illustrate conspicuous shifts in B cell subsets in the elderly, suggesting that age-related changes in B cells may contribute to immune senescence [31], [32], [33]. The discovery of a subset of B cells, termed age-associated B cells (ABCs), has drawn significant attention in recent years. In advanced fibrosis, the CD27 + B cells are decreased in the peripheral blood, causing impaired cytokine and IgG production, vaccine hyperresponsiveness, and susceptibility to bacterial infection [34]. Besides significantly lower vaccine responses and immune impairment, patients with advanced fibrosis (F3-F4) have considerably greater impairments of metabolic manifestations and features of advanced liver disease (Table 1, Table 2) as compared to low fibrosis patients (F0-F2). We reported a decrease of NK cells during NAFLD progression, mediated by insulin resistance [35]. In summary, these data suggest that advanced fibrosis in NAFLD is associated with the progression of metabolic syndrome and immune impairment that weaken the vaccine response.

**Table 2:** Laboratory data of advanced liver disease of the whole study group (Total), and two subgroups according to anti-S titers, strong responders (anti-S ≥ 200 AU/mL, N = 93) and weak responders (anti-S < 200 AU/mL, N = 64). Table 2a shows hematologic demographics and Table 2b biochemical analyses. A P-value of ≤ 0.05 is considered significant.

Table-2a				
	**Total** (N = 157)	**anti-S ≥ 200** (N = 93)	**anti-S < 200** (N = 64)	**P**
**WBC** (10E9/L)	**6.5 ±** 2.2	**6.8 ±** 2.2	**6.2 ±** 2.1	**0.053**
**Hgb** (GR%)	**13.4 ±** 1.8	**13.7 ±** 1.7	**12.9 ±** 1.8	**0.004**
**PLT** (10E9/L)	**214.1 ±** 99.0	**226.1 ±** 87.2	**196.8 ±** 112.3	**0.043**
**INR** (Ratio)	**1.1 ±** 0.2	**1.0 ±** 0.1	**1.1 ±** 0.2	**0.004**
**PTT** (Seconds)	**29.7 ±** 5.9	**29.5 ±** 6.1	**29.9 ±** 5.8	0.367
**Fibrinogen** (%)	**388.5 ±** 118.3	**414.2 ±** 109.9	**356.3 ±** 123.9	0.074

Table-2b				
	**Total** (N = 157)	**anti-S≥200** (N = 93)	**anti-S<200** (N = 64)	**P**
**Na** (mmol/L)	**137.7 ±** 12.5	**136.9 ±** 16.4	**138.7 ±** 3.1	0.209
**Creatinine** (mmol/L)	**70.7 ±** 24.9	**67.6 ±** 16.4	**74.7 ±** 32.7	**0.056**
**ALT** (U/L)	**50.5 ±** 49.6	**52.2 ±** 40.3	**48.0 ±** 61.0	0.315
**AST** (U/L)	**43.3 ±** 32.2	**40.3 ±** 22.5	**47.6 ±** 42.4	0.099
**GGT** (U/L)	**107.5 ±** 202.0	**72.6 ±** 71.5	**149.3 ±** 284.9	**0.022**
**ALP** (U/L)	**104.1 ±** 65.4	**102.9 ±** 66.8	**105.8 ±** 64.0	0.401
**Total Bilirubin** (umol/L)	**12.8 ±** 9.0	**12.4 ±** 8.5	**13.4 ±** 9.7	0.276
**Albumin** (gr/L)	**40.5 ±** 8.5	**41.9 ±** 5.4	**38.7 ±** 11.1	**0.020**
**Vitamin D** (NG/ML)	**33.7 ±** 23.1	**31.5 ±** 20.7	**35.3 ±** 25.0	0.297

**Table 3:** Data associting advanced fibrosis with lower anti-S titers using noninvasive tools. Pre-vaccination Fib-4 analysis, available in 131 cases (Table 3a), presents mean Fib-4 values and rates of advanced fibrosis (defined as Fib-4 < 2.67). Pre-vaccination Fibroscan was available for analysis in 72 cases. The mean elastography (E) and rates of advanced fibrosis, defined as E > 8.5 kPa or E > 12 kPa, is shown in Table 3b. The mean CAP readings and rates of advanced steatosis (S3), defined as > 331 dB/m, are shown in Table 3c.

	**Total (N = 131)**	**anti-S ≥ 200 (N = 77)**	**anti-S < 200 (N = 54)**	**P**
**Fib-4**	**2.4 ±** 2.6	**1.7 ±** 1.5	**3.4 ±** 3.3	**<0.0001**
**Fib-4 > 2.67**	**23.7** %	11.7 %	**40.7** %	**<0.0001**

	**Total (N** = **72)**	**anti-S≥200 (N** = **41)**	**anti-S<200 (N** = **31)**	**P**

**E (kPA)**	**11.3 ±** 9.3	**9.2 ±** 4.2	**13.6 ±** 12.4	**0.016**
**E >8.5 kPa**	**55.0** %	47.6 %	**65.8** %	0.052
**E >12 kpa**	**31.3** %	23.8 %	**42.1** %	0.041

	**Total (N** = **72)**	**anti-S≥200 (N** = **41)**	**anti-S<200 (N** = **31)**	**P**

**CAP (dB/m)**	**296.4 ±** 62.4	**296.9 ±** 57.0	**295.9 ±** 69.8	**0.475**
**CAP < 294 (S0)**	**41.7** %	46.3 %	**41.9** %	0.357
**CAP>331 (S3)**	**31.4** %	22.0 %	**41.9** %	0.035

Higher levels of all immune markers were correlated with a reduced risk of symptomatic infection. In the SIREN study, a previous history of SARS-CoV-2 infection was associated with an 84 % lower risk of infection. This study suggests that being seropositive for SARS-CoV-2 through natural infection protects robustly from asymptomatic and symptomatic reinfection [36]. Vaccine efficacy of 80 % against symptomatic infection with a majority of the Alpha (B.1.1.7) variant of SARS-CoV-2 was achieved with 264 (95 % CI: 108, 806) binding antibody units (BAU)/ml, 506 (95 % CI: 135, not computed (beyond data range) (NC)) BAU/ml for anti-spike and anti-RBD antibodies, 26 (95 % CI: NC, NC) international unit (IU)/ml and 247 (95 % CI: 101, NC) normalized neutralization titers (NF_50_) for pseudovirus and live-virus neutralization, respectively. Immune markers did not correlate with asymptomatic infections at the 5 % significance level. These data can be used to bridge to new populations using validated assays and extrapolate efficacy estimates to new COVID-19 vaccines [37]. In the current study, the cutoff of 200 AU/ml was considered to differentiate weak and strong responders according to earlier observation and based on the current study Pareto chart plots (Fig. 3). The weak response was observed in 40.7 % of the study population.

The SIREN study and others [38] reported that a short-term post-vaccination follow-up showed similar protection among weak and strong responders. However, weak and strong responses might be meaningful for longer follow-up, with the median protective effect observed seven months following primary infection. The fact that mutants affect vaccinated individuals suggests that weak rather than strong responders are susceptible. Weak responders should prioritize for third vaccination five months following the second one.

As the post-vaccine screening for antibody titers is considered not cost-effective in many countries, we propose hepatic fibrosis as a biomarker for identifying high-risk populations with a weak vaccine response to be prioritized for third vaccine consideration. Since advanced fibrosis by histology predicts a weaker vaccine response, we assessed noninvasive tools as a simpler biomarker. Both calculated Fib-4 and measured Fibroscan elastography were significantly higher in weak responders than strong responders (Table 3); these data were consistent with histological Fibrosis staging. Therefore, even simple, noninvasive tools can identify cases at risk for weak vaccine response and offer them a third vaccine five months after the second one.

Three doses of an mRNA SARS-CoV-2 vaccine were suggested for solid-organ transplant recipients [39]. In Israel, the adult population started to get three doses in July 2021 to overcome vaccine failure and mutations. In an Israeli study involving participants who were aged 60 or older and had received two doses of the BNT162b2 vaccine at least five months earlier, the researchers found that the rates of confirmed SARS-CoV-2 and severe illness were substantially lower among those who received a booster (third) dose of the BNT162b2 vaccine. Our findings suggest that older NAFLD patients with severe liver disease who received the two doses of Pfizer's BNT162b2 might be at higher risk for low SARS COV-2 protection. The noninvasive prognostic tools (Fib-4 and Fibroscan elastography) can predict the likelihood of lower post-vaccination serology.

As the current study is retrospective, major bias variables may arise from the time intervals of biopsy to vaccine and vaccine to serum serology tests. The biopsy to vaccine interval was 2.4 ± 2.4 years in the anti-S titers ≥ 200 AU/ml subgroup, significantly lower than the 4.1 ± 4.6 years in the anti-S titers < 200 AU/ml subgroup (p = 0.002). This difference also correlated with the discussed age differences. Since the liver histology of older patients does not improve with time, we assume that this interval difference does not affect the conclusion. Updated noninvasive fibrosis assessments (by Fib-4 in all cases and Fibroscan in some of them) were in line with the presented results and conclusion. The vaccine to serum serology tests intervals were 33.8 ± 30.2 days (range 7–173) in the anti-S titers ≥ 200 AU/ml subgroup, significantly lower (p < 0.0001) than the 58.8 ± 37.6 days (7–157) in the anti-S titers ≥ 200 AU/ml subgroup. Although a titer decline is reported after 4–6 months, we don't think the current approximate three-week interval explains the response differences. Athough we did not assess functional neutralizing antibodies (Nabs) in this study, there found a correlation between serum SARS-CoV-2 neutralizing antibodies and general antibodies. Neutralizing antibodies are a specific type of antibodies that can bind to and neutralize the virus, preventing it from infecting cells. General antibodies, also known as total antibodies or IgG antibodies, are a broader category that includes neutralizing antibodies and other types of antibodies produced in response to the virus. It’s important to note that the presence of neutralizing antibodies doesn’t guarantee immunity, as other aspects of the immune response, such as T-cell response, also play a crucial role in providing protection against the virus [40]. Moreover, Morales-Núñez et al. review the essential concepts of Nabs, examining their concept, mechanisms of action, production, and the techniques used for their detection; as well as presenting an overview of the clinical use of antibodies in COVID-19. They demonstrate that specific assays for have varied and, thus, are not directly comparable. Most of the studies have reported a good humoral response after a few days post-vaccination, but Nabs tend to decrease over time. However, memory B cells can rapidly deploy more antibodies in a re-exposure to the virus, and this is also true for T cells, which can attack already infected cells. In a Japanese cohort, comorbidities, such as diabetes, obesity, hypertension, dermatitis, and being overweight, have not been associated with seronegativity or low production of neutralizing antibodies, but kidney and liver diseases have been associated with a lower humoral response. It is possible that this poor response is linked to alterations in the immune system in renal disease, as uremia is associated with a state of immune dysfunction characterized by immunodepression [41].

In summary, we recommend that NAFLD patients with advanced fibrosis should consider the third vaccination. Moreover, we suggest evaluating these patients for fibrosis using noninvasive methods to prioritize for the third vaccination. More studies are necessary to understand the relationship between the severity of liver disease and SARS-CoV-2 post-vaccination infection.

## Declaration of Competing Interest

The authors declare that they have no known competing financial interests or personal relationships that could have appeared to influence the work reported in this paper.

## Data Availability

The data that has been used is confidential.
